# Development of a Work Climate Scale in Emergency Health Services

**DOI:** 10.3389/fpsyg.2018.00010

**Published:** 2018-01-22

**Authors:** Susana Sanduvete-Chaves, José A. Lozano-Lozano, Salvador Chacón-Moscoso, Francisco P. Holgado-Tello

**Affiliations:** ^1^Departamento de Psicología Experimental, Universidad de Sevilla, Seville, Spain; ^2^Departamento de Psicología, Universidad Autónoma de Chile, Santiago de Chile, Chile; ^3^Departamento de Metodología de las Ciencias del Comportamiento, Universidad Nacional de Educación a Distancia, Madrid, Spain

**Keywords:** work climate, emergency health services, mixed methods, content validity, reliability, construct validity, criterion validity

## Abstract

An adequate work climate fosters productivity in organizations and increases employee satisfaction. Workers in emergency health services (EHS) have an extremely high degree of responsibility and consequent stress. Therefore, it is essential to foster a good work climate in this context. Despite this, scales with a full study of their psychometric properties (i.e., validity evidence based on test content, internal structure and relations to other variables, and reliability) are not available to measure work climate in EHS specifically. For this reason, our objective was to develop a scale to measure the quality of work climates in EHS. We carried out three studies. In Study 1, we used a mixed-method approach to identify the latent conceptual structure of the construct *work climate*. Thus, we integrated the results found in (a) a previous study, where a content analysis of seven in-depth interviews obtained from EHS professionals in two hospitals in Gibraltar Countryside County was carried out; and (b) the factor analysis of the responses given by 113 EHS professionals from these same centers to 18 items that measured the work climate in health organizations. As a result, we obtained 56 items grouped into four factors (work satisfaction, productivity/achievement of aims, interpersonal relationships, and performance at work). In Study 2, we presented validity evidence based on test content through experts' judgment. Fourteen experts from the methodology and health fields evaluated the representativeness, utility, and feasibility of each of the 56 items with respect to their factor (theoretical dimension). Forty items met the inclusion criterion, which was to obtain an Osterlind index value greater than or equal to 0.5 in the three aspects assessed. In Study 3, 201 EHS professionals from the same centers completed the resulting 40-item scale. This new instrument produced validity evidence based on the internal structure in a second-order factor model with four components (*RMSEA* = 0.079, *GFI* = 0.97, *AGFI* = 0.97, *CFI* = 0.97; *NFI* = 0.95, and *NNFI* = 0.97); absence of Differential Item Functioning (DIF) in 80% of the items; reliability (α = 0.96); and validity evidence based on relations to other variables, specifically the test-criterion relationship (ρ = 0.680). Finally, we discuss further developments of the instrument and its possible implications for EHS workers.

## Introduction

One of the main priorities of modern organizations is fostering a positive work climate because it promotes greater productivity, satisfaction, stability, and commitment to the organization (Lozano-Lozano et al., 2013; Lee et al., 2016; Meneghel et al., 2016). There are three main types of definitions for the *work climate* construct: (1) those based on objective and structural characteristics of organizations (Schneider et al., 2013), (2) those that emphasize individual psychological features (Zadow et al., 2017), and (3) those focused on both organizational and individual levels. This last perspective emphasizes workers' perception of the structure and processes occurring in work groups (Schulz et al., 2017). This work is based on the third group of definitions because it is the most frequently used and the most complete, as it considers both organizational and individual points of view.

Independent of the type of definition, there is no consensus on what the main components that form the construct *work climate* are. In this sense, we find that measurement instruments are based on various numbers of components as follows: **three:** clarity, support and challenge (Stringer, 2002; Perry et al., 2005) and job satisfaction, organizational commitment, and motivation to continue working (Zacher and Yang, 2016); **four**: authority, efficiency, innovation, and adaptation (Payne and Mansfield, 1978); **five**: communication, work conditions, job involvement, self-realization, and supervision (Torres and Zegarra, 2015) and culture, climate, burnout, engagement, and psychosomatization (Uribe-Prado et al., 2015); **six**: organizational clarity, rewards, decisions, leadership, social interaction, and opening (Gómez, 2004); **seven**: ability, recognition, internal organization, satisfaction, information received, knowledge of management, and goals and management responsiveness (García et al., 2010); **eight**: authority, motivation, communication, influence, decision, planning, control, and performance (Likert, 1967) and compensation and justice, teamwork, quality and effectiveness, communication, environmental sustainability, trust, security, and support (Zenteno and Durán, 2016) and relationships, management style, sense of belonging, remuneration, availability of resources, stability, clarity and consistency in management, and shared values (Fernández-Argüelles et al., 2015); **nine**: structure, responsibility, reward, challenge, relationships, cooperation, standards, conflict, and identity (Litwin and Stringer, 1968); **ten**: involvement, cohesion, support, autonomy, organization, pressure, clarity, control, innovation, and comfort (Moos and Insel, 1974); and **twelve**: conflict, equipment, failure, cohesion, autonomy, management, stress, cooperation, social life, lack of shared social life, logging, and marginality (Delgado-Sánchez et al., 2006) and relationship with the boss, work environment, desire for changes, work satisfaction, capacity for making decisions, tolerance, communication and support, opportunity for training, flexibility of schedules, satisfaction with benefits, resources, and stress (Rojas et al., 2011).

The disparity of the definitions of the construct *work climate* inspires the use of diverse instruments to measure it (e.g., Insel and Moos, 1974). Additionally, there are other factors that increase the variability of the instruments to measure work climate: (1) the different levels of specification of work climate, such as an overwork climate (Mazzetti et al., 2016) or competitive work group climate (Fletcher and Nusbaum, 2010); and (2) the adaptation of instruments to specific work contexts and professions, such as civil servants (Popoola, 2016).

In the specific area of health services, we found some consolidated models, such as that proposed by Perry et al. (2005), where a work climate is understood as a part of the organization mainly generated by individual human behavior and interactions. Each work team has its own climate defined as the quality of the internal environment experienced by its members that influences their behavior. It is said that a change in the work climate of an organization implies changes in other aspects in that organization, such as the effectiveness or the quality of the care given to patients (Castaneda and Scanlan, 2015; Safi et al., 2016).

When establishing models of work climate in health services into specific components, we find a huge disparity in aspects such as work satisfaction, which is understood as the feeling of contentment by health workers with their job; this aspect correlates negatively with absenteeism and professional abandonment (Mendoza-Llanos, 2015); interpersonal relationships (the way the different components of a work group relate), productivity (the degree to which the work group is able to achieve their professional goals efficiently) and performance of the health staff (performing daily tasks), components that are positively correlated (Brown and Calnan, 2016); or commitment (level of involvement) established with the patient to achieve their professional goals (Chiang et al., 2017).

Referring to the tools used to measure the work climate in health services, we find several different instruments (Elmi et al., 2017), some of which are specific to concrete professions, such as nursing (Olsen et al., 2017) or anesthesiology (Rama-Maceiras et al., 2012), and certain fields such as mental health (Ehrhart et al., 2014).

There are substantial differences in the characteristics of work in the various departments of hospitals. In many cases, emergency health services (EHS) establish initial contact with the patient (Gill et al., 2017) and serve a large number of patients (Hunt et al., 2006) who improperly use the service on some occasions (Carret et al., 2007) and, in general, have a high incidence of morbidity (Billings and Raven, 2013). EHS are usually structured as three main areas: (1) the emergency department, an area for the triage and prioritization of care needs, observation and assessment of the patient, and referral to other hospital services (Godoi et al., 2016); (2) the intensive care unit, which usually has an isolation zone where healthcare professionals care for patients who, due to serious health problems, require critical care (Leung and Gomersall, 2016); and (3) the emergency ambulance service, which is a mobile unit where the emergency has occurred and, in some cases, avoids transport to the overloaded emergency departments (Wankhade and Mackway-Jones, 2015). Patients who receive care in the EHS are in extremely serious condition, so workers suffer from a high degree of responsibility and consequent stress (Estryn-Behar et al., 2011). They are subject to a number of external pressures, such as the need for short waiting times, and they experience detrimental impacts to their quality of life (including their own health) due to shift work (Vedaa et al., 2016), even occasionally suffering from posttraumatic stress disorder (Arora et al., 2013; Bragard et al., 2015) because of their daily experience with situations requiring critical decisions in a matter of seconds (Borg Xuereb et al., 2016). It has been verified that emergency personnel work with higher levels of stress than other health professionals and that they are a unique population with greater autonomy (Johnston et al., 2016). In this context, it is necessary to design an instrument adapted to these specific characteristics.

On the one hand, an adequate work climate in EHS allows workers to successfully share the common purpose and responsibility of professional teams, where each member clearly understands their role and combines their skills and knowledge to provide better care for their patients (Ajeigbe et al., 2013). On the other hand, an inadequate work climate in this context can cause significant stress in professionals (Laposa et al., 2003) and personal dissatisfaction, which can have an impact on the quality of health care provided (Hooper et al., 2010), increase the perception of fatigue and distress at work (Adriaenssens et al., 2011), and affect labor productivity (Engelen et al., 2016).

Measuring a work climate would be useful as a first step before acting to improve it to detect weaknesses in the work group that need strengthening. Additionally, in an indirect way, the work climate affects other important variables, such as alcohol consumption in workers (Carreño et al., 2006); safety climate (Sexton et al., 2016); motivation (Li et al., 2016); patient outcomes and nurses' occupational health (Taylor et al., 2011); and job involvement, effort, and performance (Brown and Leigh, 1996).

Some scales to measure work climate in EHS are already available in the literature (Davenport et al., 2007). However, such scales show poor psychometric properties and present only results about their reliability and, in some cases, validity evidence based on relations to other variables (Biggs et al., 2016). We also found proposals for scales that, in addition to reliability, provide data regarding validity evidence based on internal structure, such as the Safety Attitudes Questionnaire (Sexton et al., 2016) applied in different contexts (Patterson et al., 2010), but they do not provide data about validity evidence based on test content or relations to other variables. Other scales applied to EHS propose to measure something other than work climate, such as the Perceptions of Safety Climate and Adherence to Safe Work Practices (Eliseo et al., 2012), which aims to measure safety perception in emergency room conditions, or those designed exclusively to measure the work environment for nursing staff (Swiger et al., 2017).

Due to the poor consensus in the definition and measure of work climate and due to the fact that there is no specific instrument to measure this construct in EHS with adequate psychometric properties, the aim of this study was to develop an instrument to measure the quality of the work climate in EHS specifically. For this purpose, we carried out our research in three stages (American Educational Research Association et al., 2014): (1) we identified the latent conceptual structure of *work climate* using a mixed-method approach based on the information obtained from in-depth interviews using the grounded theory (Lozano-Lozano et al., 2013) and factor analysis; (2) we presented validity evidence of the resultant instrument based on the test content through experts' judgment; and (3) we assessed the psychometric properties of the final version of the instrument: we carried out studies on reliability; validity evidence based on internal structure, specifically Confirmatory Factor Analysis (CFA) and Differential Item Functioning (DIF); and validity evidence based on relations to other variables, specifically test-criterion relationships.

## Study 1: identification of the latent conceptual structure of work climate: a mixed-method approach

### Method

#### Participants

One hundred thirteen EHS workers from two different hospitals in Gibraltar Countryside County, chosen by incidental sampling, participated voluntarily. The inclusion criteria for the sample selection integrated (a) currently working in EHS when the study was carried out, and (b) having worked in EHS for at least 6 months. In total, 59.2% were women and 40.8% were men (age: *M* = 37.68 and *SD* = 8.79). Fifty percent were nurses, 22.1% were nursing assistants, 21.2% were doctors, 5.3% were orderlies, and 1.4% were administrative officers; 59.3% worked in the emergency department, 37.2% in the critical care unit and 3.5% in the emergency ambulance service. Concerning the type of contract, 36.1% had an indefinite contract, 34.1% had an interim contract, 27.8% had a temporary contract, and 2% had a contract for work and services. They had a mean of 12.83 years of experience in their profession (*SD* = 7.97); 51% had more than 5 years of experience in their profession, 21.1% had 2–5 years, and 23.8% had <2 years of experience. They had a mean of 9.38 years of experience in the current workplace (*SD* = 8.48); 52.4% had more than 5 years of experience, 28.8% had 2–5 years of experience, and 18.8% <2 years of experience.

#### Instruments

We used a list of 38 items to measure work climate in EHS specifically, which were obtained from a previous study (Lozano-Lozano et al., 2013). To produce this list, all EHS workers in the two hospitals in Gibraltar Countryside County (including doctors, nurses, nursing assistants, orderlies, administrative officers, security stuff and caretakers) were asked to voluntarily participate in answering in-depth individual interviews related to aspects such as the operation of the service, the organization, job satisfaction, the needs of the service, communication, productivity, relationship with authorities, conflicts and their resolutions, innovation and training. Eighteen EHS professionals responded: 9 doctors, 5 nurses, and 4 nursing assistants. After excluding the interviews that did not provide additional information to the built category system, the sample was formed by four doctors and three nurses. The inductive-deductive process proposed by Strauss and Corbin (1998) was followed to extract the information gathered from these interviews. Supplementary Table 1 presents the list of 38 items and their origins (codes and coding families).

Additionally, the questionnaire we used to measure the work climate in EHS was formed by 18 items rated from 1 (not at all) to 5 (to a great degree) on a five-point rating scale, available in Perry et al. (2005) to measure work climate in organizations in general, which was translated into Spanish according to the International Test Commission Guidelines for translating and adapting tests (International Test Commission, 2005; Barbero-García et al., 2008). Specifically, the following back-translation method was applied to the original English version: (a) the original version was translated into Spanish by a bilingual three-component expert group, (b) the new version was again translated into English by another bilingual translator who was not among those who formed the expert group in the first stage, and (c) the discrepancies that arose were discussed and the appropriate corrections of the new version were made. Table 1 presents the items in English. The Spanish translation is available in Supplementary Data 1. We chose these specific items for three main reasons (Perry et al., 2005): (1) the authors based their construction on a consolidated theoretical model (Stringer, 2002); (2) they presented evidence of adequate reliability, construct validity, and invariance across gender, management status, and educational level; and (3) although they are context independent, their validation was carried out in a public health organization (similar to our context, the EHS).

**Table 1:** Factor weights of the questionnaire to measure work climate (obtained from Perry et al., 2005).

**Items**	**F1**	**F2**	**F3**	**F4**
1. We take pride in our work	0.761			
2. Our work group is known for quality work	0.683			
3. We have a common purpose	0.599			
4. We seek to understand the needs of our clients	0.585			
5. We readily adapt to new circumstances	0.571			
6. We strive to achieve successful outcomes	0.487			
7. We understand the relevance of the job of each member in our group		0.803		
8. We understand each other's capabilities		0.768		
9. We strive to improve our performance		0.623		
10. We pay attention to how well we are working together		0.618		
11. We are recognized for our individual contributions			0.740	
12. We have the resources we need to do our jobs well			0.722	
13. We have a plan that guides our activities			0.617	
14. We participate in the decisions of our work group			0.528	
15. Our work group is productive			0.511	
16. Our work is important				0.824
17. We develop our skills and knowledge				0.719
18. We are clear about what is expected in our work				0.511

#### Procedure

##### Survey methodology

We contacted the management of the EHS of Gibraltar Countryside County (two different hospitals) and presented the reasons for the research and the characteristics of the study. Once we obtained the corresponding authorization, the 18-item questionnaire obtained from Perry et al. (2005) was administered to the staff of the EHS (250 participants). To ensure anonymity, the workers completed the questionnaire in a room in the hospital and, once finished, they deposited it in a ballot box.

With the data gathered, we carried out an exploratory factor analysis (EFA) (Pérez-Gil et al., 2000). SPSS 24 was used to store the data; PRELIS and LISREL 9.2 were used to carry out the data analysis. First, we created a polychoric correlation matrix between all of the variables that came into the analysis (Holgado-Tello et al., 2010). Second, we checked whether such a matrix met the assumptions necessary to be able to develop an EFA (Yela, 1957) by calculating the Kaiser-Meyer-Olkin (KMO) test and Bartlett's test of sphericity. Third, we performed an EFA using a principal components factor extraction and Kaiser's varimax method for orthogonal rotation.

##### Mixed-method approach

With the survey methodology, we calculated the factor structure of the 18 items obtained from Perry et al. (2005) in a specific EHS context. To obtain more detailed information about the important aspects that form a work climate in this specific context, we integrated these 18 items considering their factor structure and the 38 items obtained from in-depth interviews (Lozano-Lozano et al., 2013) considering their coding families.

The reliability of the assignment item—factor was checked using SPSS 24 in two ways: (a) by intracoder, where one author, JALL, completed the assignment and repeated the same task after 1 month; and (b) by intercoder, where two different authors, SCM and SSC, independently performed the same task. In both cases, Cohen's kappa (κ) coefficient was calculated.

### Results

#### Survey methodology

Of the potential 250 participants, 113 answered, which is a participation rate of 45.2%. Assumptions to develop an EFA were accepted: the KMO was 0.851, and Bartlett's test of sphericity resulted in $\chi_{\left(153\right)}^{2}$ = 804.174, *p* < 0.001.

The EFA provided a four-factor solution that explained 61.33% of the common variance. Factor 1 (F1) *Work satisfaction* produced an eigenvalue of 6.57 and explained 36.54% of the common variance. F2 *Productivity/achievement of aims* produced an eigenvalue of 1.93 and explained 10.79% of the common variance. F3 *Interpersonal relationships* produced an eigenvalue of 1.34 and explained 7.44% of the common variance. Finally, F4 *Performance at work* produced an eigenvalue of 1.17 and explained 5.47% of the common variance.

Table 1 shows the highest factor loading for each item. All values were higher than 0.48.

#### Mixed-method approach

Table 2 presents the scale used to measure the work climate in EHS after combining the results obtained with the in-depth interviews and the survey methodology. The assignment item—factor produced an intracoder reliability κ of 0.922 and an intercoder reliability κ of 0.879. Both results were considered adequate.

**Table 2:** Resultant work climate scale in emergency health services after finishing the mixed-method approach (Study 1, in-depth interviews—survey) and Osterlind indexes obtained in Study 2, validity evidence based on test content.

**Item**	**R**	**U**	**F**
**FACTOR 1. WORK SATISFACTION**			
*1. We take pride in our work*	0.50	0.59	0.55
*2. Our work group is known for quality work*	0.58	0.75	0.77
*3. We have a common purpose*	0.58	0.67	0.55
*4. We seek to understand the needs of our clients*	0.79	0.59	0.64
*5. We readily adapt to new circumstances*	0.63	0.54	0.55
*6. We strive to achieve successful outcomes*	0.96	0.88	0.73
7. We have the experience to do our work well	0.79	0.77	0.65
8. Our workday is adequate to develop our work	0.68	0.63	0.50
9. Time to perform my work well	0.54	0.64	**0.45**
10. Time to attend to our users	0.58	0.55	**0.31**
11. Similar amount of work as in other centers	**0.17**	**0.18**	**0.25**
12. Good relations with the other services	0.75	0.60	0.50
13. We have a good relationship with our patients	**0.46**	**0.32**	**0.40**
14. Good relationship with relatives	**0.14**	**0.09**	**0.33**
15. Patients have good relationship with hospital	**0.46**	**0.30**	**0.33**
**FACTOR 2. PRODUCTIVITY/ACHIEVEMENT OF AIMS**			
*16. Relevance of the job of each member*	0.58	0.73	0.50
*17. We understand each other's capabilities*	0.71	0.63	0.68
*18. We strive to improve our performance*	0.63	0.55	**0.35**
*19. We pay attention to how well we are working*	0.68	0.65	**0.35**
20. We have the necessary infrastructure	0.71	0.63	0.68
21. We receive the necessary training	0.67	0.88	0.60
22. The characteristics of our service are appropriate	0.63	0.70	0.61
23. Our service works correctly	0.71	0.90	0.56
24. Our work group is known for its productivity	0.79	0.79	0.70
25. Our work is guided by protocols for action	**0.32**	**0.15**	0.60
26. We feel motivated doing our work	0.79	0.79	0.50
27. The merit of our good job is recognized	0.71	0.73	0.50
28. Our colleagues value our profession	0.77	0.73	0.60
29. We are appreciated for the work we do	0.83	0.86	0.60
30. Our specialization is recognized	0.75	0.68	0.55
31. Our expectations have been fulfilled	0.83	0.80	0.60
32. Our type of patient fits with the service	0.75	0.60	0.61
33. We always attend to patients in emergencies	**0.35**	**0.45**	**0.44**
34. We know our patients' characteristics	0.59	0.70	0.50
35. We coordinate with the other hospital services	0.65	0.50	0.78
**FACTOR 3. INTERPERSONAL RELATIONSHIPS**			
*36. We are recognized for our individual contributions*	0.71	0.82	0.55
*37. We have the resources we need*	**0.42**	**0.45**	0.70
*38. We have a plan that guides our activities*	0.50	0.55	0.60
*39. We participate in the decisions of our group*	0.67	0.70	0.60
*40. Our work group is productive*	**0.33**	**0.32**	0.50
41. We have good communication within the group	0.79	0.82	0.65
42. Good relationship between members	0.83	0.86	0.61
43. I feel comfortable with my work group	0.82	0.80	0.55
44. I have good personal relationships	0.67	0.64	0.50
45. We work in a good work group climate	0.67	0.77	0.61
46. Before entering, I knew some of its members	**0.33**	**0.10**	**0.40**
47. We know how to manage conflict	**0.33**	**0.40**	0.50
48. Good relationship with our work group chief	0.71	0.77	**0.40**
**FACTOR 4. PERFORMANCE AT WORK**			
*49. Our work is important*	0.92	0.86	0.70
*50. We develop our skills and knowledge*	0.83	0.82	0.60
*51. We know what is expected in our work*	0.79	0.73	0.65
52. I know my professional shortcomings	0.54	0.64	0.50
53. We know the functions of the members	0.75	0.75	0.72
54. Our patients fit the specialty of our service	0.79	0.73	0.70
55. We know our shortcomings as a group	0.75	0.60	0.50
56. We can make proposals to improve our work	0.55	**0.25**	**0.44**

*Some items are presented in short format. The full version is available in Supplementary Data 2. N = 14. Items from the survey method are marked in italics. The rest come from the in-depth interviews. R, representativeness; U, utility; F, feasibility. Values under the cut point (<0.5) are marked in bold*.

F1 *Work satisfaction* refers to feelings evoked in workers by their job and their conditions: self-confidence due to the experience, the adequacy of the workday or time for each patient, contentment with relationships with other professionals outside the group and patients and their relatives, pride, success, cohesion, or nervousness facing new circumstances. F1 is formed using 15 items, six from the survey method (items 1–6) and nine from the in-depth interviews (items 7–15, corresponding to items 1–9 in Supplementary Table 1).

F2 *Productivity/achievement of aims* refers to the perception of workers having everything they need to do their job or, on the contrary, lacking what they need to achieve their goals: understanding of the relevance, capabilities, and specialization of others; the value of working in a group; motivation and fulfillment of expectations; recognition of their work as a group; self-improvement; infrastructure; training and the characteristics and functioning of their service; patients' characteristics fitting with their specialization and knowledge of such characteristics; protocols; and coordination with other hospital services. F2 is formed by 20 items, four from the survey method (items 16–19) and 16 from the in-depth interviews (items 20–35, corresponding to items 10–25 in Supplementary Table 1).

F3 *Interpersonal relationships* refer to the feelings when workers relate to other members of the group and aspects that influence such feelings: the quality of the communication, their relationship, the level of comfort, their friendship, their conflicts, being recognized for their individual contributions, having the resources they need, following a plan, participating in decision-making, and productivity. Thirteen items, five from the survey method (items 36–40) and eight from the in-depth interviews (items 41–48, corresponding to items 26–33 in Supplementary Table 1) form F3.

Finally, F4 *Performance at work* includes everything related to the development of workers' job placement: the perceived importance of their job and capacity to decide how to improve their performance; the skills and knowledge they use; and their knowledge of their tasks and others' tasks, their individual and group limitations, and their patients. F4 is formed by eight items, three from the survey method (items 49–51) and five from the in-depth interviews (items 52–56, corresponding to items 34–38 in Supplementary Table 1).

## Study 2: validity evidence of the resultant instrument based on test content

### Method

#### Participants

We requested 27 experts' collaboration. The inclusion criterion was to have more than 3 years of experience in social and health sciences methodology and/or in EHS as a health professional. Fourteen experts answered, which is considered a moderate number of participants (10 ≤ *N* ≤ 30) for a content validity study (Prieto and Muñiz, 2000).

Eight experts (57.1%) were men and six (42.9%) were women. Their mean age was 48.83 (*SD* = 8.99). Regarding their professions, nine (64.3%) were professors specializing in methodology, design, psychometrics and/or data analysis, four (28.6%) were physicians from EHS, and one (7.1%) was a senior technician in clinical analysis and emergency and intensive care medicine. They were in their professions a mean of 23.67 years (*SD* = 9.46).

#### Instruments

The questionnaire used to obtain validity evidence based on test content through experts' judgment was composed of the 56 items obtained in the previous mixed-method study ordered in the four factors or dimensions found (see Supplementary Data 2). Each item presented three five-point Likert scales (Sanduvete-Chaves et al., 2013) to measure their representativeness (R), utility (U), and feasibility (F) (Chacón-Moscoso et al., 2016). Additionally, there was a final open-format question to receive comments for improvements for the proposed scale. Supplementary Data 3 presents the Spanish version of the questionnaire used to obtain validity evidence based on test content, which was completed by native Spanish speakers.

#### Procedure

The questionnaire to obtain validity evidence based on test content was sent by e-mail to 27 experts. After the third request, a total of 14 experts gave their answers. Anonymity was assured.

The Osterlind index of congruence (1998) was used to quantify the consensus between experts regarding the adequacy item—factor (theoretical dimension) (Glück et al., 2013). The formula used was

$$\begin{array}{l} I_{ik}=\frac{\left(N-1\right)\overset{n}{\underset{j=1}{\sum }}X_{ijk}+N\overset{n}{\underset{j=1}{\sum }}X_{ijk}-\overset{n}{\underset{j=1}{\sum }}X_{ijk}}{2\left(N-1\right)n} \end{array}$$

where *N* = the number of dimensions of the instrument; *X*_*ijk*_ = each score given by each expert to each item referring to each aspect (R, U, and F); and *n* = the number of experts. The results could be from −1 to +1, 0 being the highest possible level of disagreement between experts. The inclusion criterion was to produce at least 0.5 (Osterlind, 1998) in the three aspects evaluated (R, U, and F).

### Results

Considering that we requested assessments from 27 experts and 14 answered, we obtained a 51.9% rate of participation.

Forty items met the inclusion criterion; 16 did not meet it. Table 2 presents the Osterlind indexes for each item and each aspect (R, U, and F). The most frequently undervalued aspect was F: from the 16 items excluded, 14 were rated under 0.5 in this aspect.

Additionally, several experts proposed a reorganization of some items with respect to its factor assignment through the open-format question: four and three experts, respectively, suggested including item 49, referring to the perceived importance of their work, and item 50, referring to the development of their skills and knowledge at work, in F1 *Work satisfaction* instead of F4 *Performance at work*. Four experts suggested including item 2, referring to the quality of work and item 3, referring to the existence of a common purpose, in F2 *Productivity/achievement of aims* instead of F1. Two experts proposed including item 51, referring to the knowledge of what is expected in their work, in F2 instead of F4 *Performance at work*; and vice versa, five experts proposed including item 17, referring to the understanding of each other's capabilities, in F4, instead of F2.

The four authors of this work considered these proposals individually. After debating, we accepted all the suggestions given by consensus, based on the adequacy item—factor (dimension), from a substantive point of view.

## Study 3: reliability and validity evidence based on internal structure and relations to other variables

### Method

#### Participants

Two hundred and one EHS professionals from the same two hospitals in Study 1 participated voluntarily. The inclusion criteria for the sample selection included (a) working in EHS when the study was carried out, and (b) having worked in EHS for at least 6 months. In total, 61.7% were women and 38.3% were men (age: *M* = 41.6 and *SD* = 10.23). Of these, 40.3% were nurses, 33.3% were doctors, 22.4% were nursing assistants, and 4% were orderlies. Sixty-seven percent worked in the emergency department, 30.5% to the critical care unit, and 2.5% to the emergency ambulance service; 44.6% had a temporary contract, 26.3% had an indefinite contract, 17.1% had an interim contract, 10.9% were residents, and 1.1% had a contract for work and services. Regarding their years of experience in their profession, they had a *M* of 15.12 (*SD* = 9.64); 49.2% had more than 5 years in their profession, 22.4% had 2–5 years of experience, and 28.4% had <2 years of experience. Regarding the number of years in their current workplace, the mean was 8.52 (*SD* = 8.31); 81.6% had more than 5 years of experience, 13.8% had 2–5 years of experience, and 4.6% had <2 years of experience.

#### Instruments

The scale used was formed via instructions for participants and the items that met the inclusion criterion in Study 2 (content validity), grouped into factors per expert suggestion (see Supplementary Data 4 for the English version and Supplementary Data 5 for the Spanish version). Each item was valued from 1 (strongly disagree) to 5 (strongly agree).

Furthermore, we added one extra omnibus item: *As a whole, the work climate of my work group is good;* in the Spanish version, *De manera global, el clima laboral de mi grupo de trabajo es bueno*; translation carried out according to the International Test Commission recommendations (International Test Commission, 2005; Barbero-García et al., 2008), which was also valued from 1 (strongly disagree) to 5 (strongly agree) and was used as a criterion in Study 3. This item was used as a criterion based on the following reasons (Holgado-Tello et al., 2015): (a) it can be considered an appropriate direct measure to relate to another indirect measure (the resulting scale) in the sense that both refer to the same construct (work climate in EHS); (b) the Likert-type scale of the item permits obtaining a linear monotonic function with respect to the attitude measured as the item characteristic curve, and individual differences in participants' attitude provoke variation in responses; and (c) no instrument with its psychometric properties tested that measures work climate in EHS was available as a criterion.

#### Procedure

The information was gathered using Google for Work applications: Drive, Forms, and Spreadsheets. The scale was administered by two procedures: (a) using a laptop with internet access in the hospital and (b) sending emails to all the EHS professionals with the link to the scale; in this case, they answered outside the work context.

SPSS 24 was used to store the data and calculate the internal consistency of the test, the average discrimination index, and validity evidence based on a test-criterion relationship and needed assumption tests. PRELIS and LISREL 9.2 were used to estimate the polychoric correlation matrix to verify bivariate normality and to carry out the CFA.

The internal consistency of the items was calculated using Cronbach's alpha coefficient, following criteria established by George and Mallery (2003). Values > 0.9 were considered excellent, 0.8–0.9 good, and 0.7–0.8 acceptable. Following criteria by Tavakol and Dennick (2011), values equal to or higher than 0.7 were considered appropriate.

The average discrimination index was also calculated: values >0.4 were considered excellent (Sabri, 2013), 0.3–0.4 good, and 0.2–0.3 adequate (Barbero-García, 1993). Obtaining adequate values in internal consistency and on the average discrimination index was considered an essential requirement before proceeding to study the factor structure.

To evaluate the factor structure of the scale, we estimated the polychoric correlations and the asymptotic variance-covariance matrix. A Pearson correlation matrix was not estimated because the items were in ordinal scales, and therefore, the responses cannot be treated as if they were quantitative because all participants situated at different points of the interval may be assigned the same score; the use of Pearson correlations for ordinal scales would undervalue the real correlations (Holgado-Tello et al., 2010). In these cases, polychoric correlations are the most consistent and robust estimator (Morata-Ramírez and Holgado-Tello, 2013).

The use of the polychoric correlation matrix is only appropriate if we previously accept the assumption of bivariate normal distribution. For this purpose, we calculated the chi-square test (χ^2^) and the percentage of tests that rejected the null hypothesis of bivariate normality for each pair of correlations, assuming a 95% confidence level and the Bonferroni correction, calculating the value of α to use in the comparison of each contrast with the formula α*/c* (α = 0.05 corresponding to a 95% confidence level and *c* as the number of contrasts [*c* = (number of items x number of items – 1)/2]. Due to the sensitivity of χ^2^ in large samples, we also calculated the root mean square error of approximation (RMSEA). We concluded that the parameter estimation was not significantly affected when RMSEA values did not exceed 0.1 (Hooper et al., 2008).

Once we tested the assumption of bivariate normal distribution, we then tested the resultant model from Studies 1 and 2 using a CFA (Bagozzi and Yi, 2012). The tested model was a second-order factor model that measures work climate in EHS formed by four factors (Table 2): F1 *Work satisfaction* (items 1–10); F2 *Productivity/achievement of aims* (items 11–30); F3 *Interpersonal relationships* (items 31–35); and F4 *Performance at work* (items 36–40).

The estimation method used was the unweighted least squares, which is appropriate for polychoric correlations and ordinal variables distributed asymmetrically (Jöreskog, 2003; Morata-Ramírez et al., 2015).

The lambda parameter corresponding to the relationship of the first item with each factor was fixed at 1 to (a) solve the problem of identification of the model and (b) establish the measurement scale of the latent variables.

The standardized factor loadings were calculated. Additionally, several fit indices were used to reach conclusions about the adequacy of the model: (a) the χ^2^ test, where the acceptance of the null hypothesis (*p* ≥ 0.05) implied a good fit of the model; (b) the consistent Akaike information criterion (CAIC) with which the model was considered appropriate when the value of the index was closer to the value for the saturated model than the independent one (the smaller the values, the better the fit) (Bandalos, 1993); (c) the root mean square error of approximation (RMSEA) (Hooper et al., 2008), where values lower than 0.05 were considered a good fit, values between 0.08 and 0.1 a reasonable fit, and values greater than 0.1 unfit (Browne et al., 1993); (d) the goodness-of-fit index (GFI); (e) the adjusted goodness-of-fit index (AGFI) (Hooper et al., 2008); (f) the comparative fit index (CFI) (Byrne, 1998); (g) the normed fit index (NFI); and (h) the non-normed fit index (NNFI) (Hoe, 2008). Indices (d)–(h) were interpreted as indicators of good fit if the values were above 0.9 (Bendayan et al., 2013).

We also studied DIF to obtain additional validity evidence based on the internal structure of the scale. EASY-DIF (González et al., 2011) was used to perform the Mantel-Haenszel procedure for ordinal items. The DIF was calculated by gender; seniority at work (0–15 years and 16–40 years of experience); age (18–42 and 43–65 years old); and type of employment relationship (eventual and permanent). The matching method used was the minimum cell frequency.

Additionally, we calculated the validity evidence based on the test-criterion relationship, correlating the global score (*X*, the sum of the scores given in the 40 items) and those obtained in each factor with the score given in the omnibus item 41: *As a whole, the work climate of my work group is good* (criterion *Y*) (Holgado-Tello et al., 2015). Previously, we tested several assumptions to check that the use of the Pearson correlation coefficient (*r*), a parametric test, was adequate: (a) normality using the Kolmogorov Smirnov test, where *p* > 0.05 implied the acceptance of the assumption (Chakravarti et al., 1967); (b) a test of linearity, where linearity *p* < 0.05 implied the acceptance of the assumption (Field, 2000); and (c) independence of errors using Durbin Watson (*d*), a statistic that ranged from 0 to 4 and was expected to produce a value close to 2; thus, values between 1.5 and 2.5 implied acceptance of the assumption (Kutner et al., 2004). If one of the three assumptions is rejected, we would opt for a non-parametric correlation test (Spearman's correlation coefficient, ρ). Both the Pearson and Spearman would be interpreted as validity evidence based on a test-criterion relationship when a *p* < 0.05 showed a statistically significant relationship between the global scores and *Y*.

### Results

#### Participation

From the potential 250 participants, 201 completed the scale, producing a participation percentage of 80.4%; in particular, 199 (79.6%) were recorded in the hospital, and two (0.8%) by email outside the work context.

#### Internal consistency

The global internal consistency was excellent (α = 0.96). Factors also produced appropriate values, all over 0.7; F1 produced a good result (α = 0.848); F2 and F3 produced excellent results (α = 0.943 and 0.907, respectively); and F4 produced acceptable, close to good results (α = 0.791).

#### Average discrimination index

The global average discrimination index produced an excellent result (*D* = 0.601), as did the specific index for each of the four factors (0.515, 0.646, 0.654, and 0.519, respectively). After testing the appropriateness of the internal consistency and the average discrimination index, we concluded that we could carry out the factor structure study.

#### Bivariate normality assumption

By having 40 items, a total of 780 correlations were obtained (40 × 39/2). The results showed that a bivariate normality assumption considering χ^2^ was accepted in 95.3% of the instances (743 correlations) (*p* = 0.05/780 = 0.00006 using the Bonferroni correction). Additionally, the RMSEA values were lower than 0.1 in 98.5% of occasions (768 correlations). These results support the use of the matrix of polychoric correlations as the basis for the factor analyses.

#### Standardized factor loadings

The standardized factor loadings (lambda) in the CFA (Table 3) were appropriate (over 0.3) for all of the items. The gamma values were high for the four factors (0.89, 0.81, 0.82, and 0.86, respectively).

**Table 3:** Standardized factors loadings obtained in the CFA.

**Item**	**F1**	**F2**	**F3**	**F4**
1. We take pride in our work	0.69			
2. We seek to understand the needs of our clients	0.64			
3. We readily adapt to new circumstances	0.51			
4. We strive to achieve successful outcomes	0.71			
5. We have experience to do our work well	0.40			
6. Our workday is adequate to develop our work	0.68			
7. Good relations with the other services	0.87			
8. Relevance of the job of each member	0.73			
9. Our work is important	0.73			
10. We develop our skills and knowledge	0.69			
11. Our work group is known for quality work		0.64		
12. We have a common purpose		0.75		
13. We have the necessary infrastructure		0.75		
14. We receive the necessary training		0.74		
15. The characteristics of our service appropriate		0.76		
16. Our service works correctly		0.76		
17. Our work group is known for its productivity		0.64		
18. We feel motivated doing our work		0.71		
19. The merit of our good job is recognized		0.73		
20. Our colleagues value our profession		0.77		
21. We are appreciated for the work we do		0.87		
22. Our specialization is recognized		0.73		
23. Our expectations have been fulfilled		0.79		
24. Our type of patient fits with the service		0.65		
25. We know our patients' characteristics		0.79		
26. We coordinate with the other hospital services		0.55		
27. We are recognized for our individual contributions		0.81		
28. We have a plan that guides our activities		0.72		
29. We participate in the decisions of our group		0.82		
30. We know what is expected in our work		0.71		
31. We have good communication within the group			0.86	
32. Good relationship between members			0.81	
33. I feel comfortable with my work group			0.87	
34. I have good personal relationships			0.84	
35. We work in a good work group climate			0.90	
36. We understand each other's capabilities				0.64
37. I know my professional shortcomings				0.64
38. We know the functions of the members				0.67
39. Our patients fit the specialty of our service				0.68
40. We know our shortcomings as a group				0.55

*Some items are presented in short format. The full version is available in Supplementary Data 4. F1, Factor 1 Work satisfaction; F2, Factor 2 Productivity/achievement of aims; F3, Factor 3 Interpersonal relationships; F4, Factor 4 Performance at work*.

#### Model fit

Although the χ^2^ test was significant, probably due to the large sample size effect, $\chi_{\left(730\right)}^{2}$ = 3807.37, *p* < *0.001*, the other fit indexes showed the adequacy of the second-order-four-factor model: *CAIC* = 2460.93 (saturated CAIC = 5347.60; independent CAIC = 39115.98); *RMSEA* = 0.079, 90% CI [0.075, 0.084]; *GFI* = 0.97; *AGFI* = 0.97; *CFI* = 0.97; *NFI* = 0.95; and *NNFI* = 0.97.

#### DIF

Thirty-two items (80% of the total) did not present any DIF; the 8 items that presented some DIF were items 2, 3, 5, 6, 18, 23, 27, and 29. For the variable gender, the items with DIF were items 3 (χ^2^ = 4.38, *p* = 0.03), 6 (χ^2^ = 8.3, *p* < 0.001), 23 (χ^2^ = 6.44, *p* = 0.01), 27 (χ^2^ = 4.73, *p* = 0.02), and 29 (χ^2^ = 6.95, *p* < 0.001). In items 3, 6 and 23, women scored higher than men, while in items 27 and 29, men scored higher than women. In seniority at work, the workers with more years in the company scored higher in items 2 (χ^2^ = 4.28, *p* = 0.03) and 5 (χ^2^ = 6.82, *p* < 0.001), while workers with fewer years of experience working scored higher in item 18 (χ^2^ = 7.27, *p* < 0.001). For the variable age, older workers scored higher in item 5 (χ^2^ = 6.07, *p* = 0.01*)* and item 6 (χ^2^ = 5.74, *p* = 0.01), while younger people scored higher in item 18 (χ^2^ = 4.47, *p* = 0.03). Finally, for type of employment relationship, workers with permanent contracts scored higher in items 5 (χ^2^ = 4.94, *p* = 0.02) and 6 (χ^2^ = 6. 78, *p* < .001), while workers with eventual contracts scored higher in item 18 (χ^2^ = 6.19, *p* = 0.01).

#### Testing assumptions before the criterion validity correlation

The normality assumption was accepted on the global scale (*Z* = 0.607, *p* = 0.855), in F1 (*Z* = 1.329; *p* = 0.058) and in F2 (*Z* = 0.693, *p* = 0.722), while it was rejected in F3 (*Z* = 1.729, *p* = 0.005), F4 (*Z* = 1.409, *p* = 0.038), and the criterion (*Z* = 3.047, *p* < 0.001). The linearity assumption was accepted in all instances when relating with *Y, X* [*F*_(1, 105)_ = 187.569, *p* ≤ 0.001]; F1 [*F*_(1, 168)_ = 88.717, *p* ≤ 0.001]; F2 [*F*_(1, 138)_ = 146.957, *p* < 0.001]; F3 [*F*_(1, 177)_ = 141,197, *p* ≤ 0.001]; and F4 [*F*_(1, 177)_ = 41,335, *p* < 0.001]. Finally, the independence of errors was accepted in all occasions for the relationship of *Y* with *X* (*d* = 1.608), F1 (*d* = 1.654), F2 (*d* = 1.634), F3 (*d* = 1.792), and F4 (*d* = 1.761). As a conclusion, although most assumptions were accepted, because there was no normal distribution in *Y*, the non-parametric test (ρ) was used to study the relationship between *Y* and *X*, and *Y* and the sum of the different factors.

#### Validity evidence based on a test-criterion relationship

Validity evidence based on a test-criterion relationship for the whole scale presented an adequate result, ρ_*XY*_ = 0.68, *p* < 0.001. The relationships between the sum of scores in the different factors and *Y* also yielded adequate results with F1 (ρ = 0.560, *p* < 0.001), F2 (ρ = 0.632, *p* < 0.001), F3 (ρ = 0.658, *p* < 0.001), and F4 (ρ = 0.430, *p* < 0.001).

## Discussion

Based on the non-consensus of the main components that form the construct *work climate* and the lack of an instrument to measure it, by being applied to EHS specifically and with adequate psychometric properties, we elaborated a scale consisting of four dimensions (work satisfaction, productivity/achievement of aims, interpersonal relationships, and performance at work) and 40 items, which presented evidence of reliability as well as validity based on test content, internal structure and relations to other variables. Supplementary Data 4, 5 present the final version of the scale ready to be used by those who are interested in the English and Spanish versions, respectively.

Thus, as a result, Study 1 (a mixed-method approach) produced 56 items grouped into the four previously mentioned components. Study 2 (validity evidence based on test content through experts' opinion) permitted refinement of the scale in accordance with the representativeness, utility, and feasibility of its items (16 were removed as a result), and six were reorganized into different factors. Finally, Study 3 (psychometric properties) presented a second-order factor model with the four previously mentioned components with adequate values for reliability and validity evidence based on internal structure and test-criterion relationships.

The four factors obtained have certain similarities to those obtained in previous studies: (a) Work satisfaction (F1) is one of the factors that form the construct work climate in models by García et al. (2010), Rojas et al. (2011), and Zacher and Yang (2016); (b) Productivity/achievement of aims (F2) appears in Payne and Mansfield (1978) with the label of efficiency and in Brown and Calnan (2016); (c) Interpersonal relationships (F3) is also a factor in the proposals given by Fernández-Argüelles et al. (2015) and Litwin and Stringer (1968); finally, (d) Performance at work (F4) is a factor also found in Likert (1967) and Brown and Calnan (2016). On the other hand, some elements that are not in previous studies have been found to be relevant to measuring the work climate in EHS, such as items to evaluate personal and group shortcomings and possible quarrels between different professions in terms of delimitation of functions according to their specialty and recognition.

We obtained four factors when compared to the four-factor model by Payne and Mansfield (1978)—authority, efficiency, innovation, and adaptation—and we find one in common: productivity/achievement of aims, which is understood as efficiency. Although the other three factors are different, some of the contents of the items of our proposal are related, e.g., item 29, *We participate in the decisions of our work group*, refers to authority; item 13, *We have the necessary infrastructure to carry out our work*, and item 14, *We receive the necessary training to carry out our tasks* are related to innovation; and finally, item 3, *We readily adapt to new circumstances*, is related to adaptation.

Additionally, given that the partial starting point of this work was 18 items proposed by Perry et al. (2005) and that 15 have been included in the last version of the proposed scale presented in Supplementary Data 4 (specifically, items 1–4, 8–12, 17, 27–30, and 36), we now compare the psychometric properties of both instruments. The reliability values were adequate in both cases: α = 0.96 in the final version of the scale proposed and α = 0.87 in Perry et al. (2005). Both instruments obtained evidence based on the internal structure, although the factors found differ substantively, where three (clarity, support, and challenge) were found in Perry et al. (2005). Both instruments obtained evidence based on the relation test—criterion: ρ_*XY*_ = 0.68, *p* < 0.001 when correlating the scale obtained and an omnibus item and *r*_*XY*_ = 0.93, *p* < 0.001 in Perry et al. (2005) after correlating their instrument with the one proposed by Stringer (2002). In this sense, because both proposals presented adequate psychometric properties, the most important contribution of the scale proposed in this work is its specification in setting; Perry et al. (2005) based their study on public health organizations, and the present work is specifically for EHS, a context with an extremely specific idiosyncrasy (Hunt et al., 2006; Carret et al., 2007; Arora et al., 2013; Johnston et al., 2016; Vedaa et al., 2016; Gill et al., 2017).

Taking into account that any process to obtain evidence is affected by the characteristics of the intervention contexts in this particular case, given the sample characteristics, the instrument was designed with the aim of measuring the global construct *quality of the work climate* (as opposed to proposals centered on a specific aspect within work climate, e.g., Fletcher and Nusbaum, 2010; Mazzetti et al., 2016) for all professionals (as opposed to works based on specific professions, e.g., Rama-Maceiras et al., 2012; Popoola, 2016; Olsen et al., 2017) who work in EHS (as opposed to other specific contexts, e.g., Ehrhart et al., 2014).

To check empirically that the items worked in an unbiased way across different groups, we analyzed their DIF. Thirty-two items (80%) did not present any DIF. The fact that 8 items (20%) presented DIF does not necessarily indicate the weakness of the instrument given that substantive reasons explain the differences (American Educational Research Association et al., 2014). Item 2, *We seek to understand the needs of our clients*, was scored higher by workers with more experience because there is evidence that confirms a direct relationship between experience and implication with the patients (Ballester-Arnal et al., 2016) and the experience and perception of patients' needs in a holistic way instead of only targeting the isolated symptom (Zamanzadeh et al., 2015). Item 3, *We readily adapt to new circumstances*, and 23, *Our expectations when we entered the working group have been fulfilled*, were scored higher by women because females have a greater ability to adapt to new situations (Catalyst, 2007) and are more conformist (Aspiazu, 2016) in work. Item 5, *We have the necessary experience to do our work well*, was scored higher by workers with more experience, those who were older and those who had permanent contracts due to a real difference in the level of the aspect measured in this item (experience). Item 6, *Our workday is adequate to develop our work*, was scored higher by women, older workers, and those with permanent contracts. Differences in gender can be explained by the fact that women tend to assign greater value and spend time most productively in the workplace since they are also involved in other types of family activities (Artazcoz et al., 2004; Eagly and Carli, 2007); and workers with more experience and permanent contracts spend less time carrying out their functions than those who are less experienced and with eventual contracts, so it is logical that the first group believes more strongly than the second one that the time they have is sufficient to perform their work. Item 18, *We feel motivated when doing our work*, was more valued by younger workers with eventual contracts because studies conclude that there is an inverse relationship between age and job stability and motivation at work (Fernández et al., 2015; Akkermans et al., 2016; Dawson et al., 2017). Finally, items 27, *We are recognized for our individual contributions*, and 29, *We participate in the decisions of our work group*, were scored higher by men because they tend to develop dominant behaviors such as autonomy, independence and decision-making and seeking individual social recognition (Eagly et al., 2004; Godoy and Mladinic, 2009).

The proposed tool can be used in EHS to measure the work climate. Apart from a global value for each worker obtained by summing the values for the 40 items, the tool can be used to detect the global average work climate in EHS, factor by factor, or by studying each item to detect weaknesses so as to implement actions (with the origin in the work colleagues or the boss) to improve them; e.g., if a worker scored item 5 low (*We have the necessary experience to do our work well)*, a workmate with more experience could start acting as a temporary mentor, or if the average of a work group in item 20 (*Our colleagues value our profession*) is low, then the boss of the group could implement strategies to improve the multidisciplinary work group conditions. In addition, by measuring the values with the scale before and after the actions implemented to improve the work climate, we can see the effectiveness of such actions (the use of inferential statistics for repeated measures would give evidence about the significance of the change).

In summary, the proposed scale can be used as a tool to diagnose the climate given in the work place and, based on this information, implement action to attempt to improve the situation (Perry et al., 2005). This can involve improvements at different levels since increasing the quality of the work climate will probably influence workers' satisfaction (Hooper et al., 2010), productivity (Brown and Leigh, 1996), interpersonal relationships (Lozano-Lozano et al., 2013), and performance (Engelen et al., 2016). Furthermore, it may act as a protective factor against alcoholism (Carreño et al., 2006), stress (Laposa et al., 2003), fatigue (Adriaenssens et al., 2011), or absenteeism (Mendoza-Llanos, 2015) in workers. Therefore, it will probably increase patients' satisfaction with the service (Hooper et al., 2010; Ajeigbe et al., 2013).

One limitation to highlight is the length of the resultant scale. Although 40 items to measure four factors is apparently not excessive, it is important to note that, currently, workers in EHS receive more patients than they can manage, so it would be difficult for them to find enough time to complete such a long instrument. Another possible limitation is the use of one item as a criterion. In this sense, it is necessary to carry out research that further develops the instrument obtained in this study.

To cover the first limitation (excessive length of the scale), we will shorten the scale without excluding relevant information to measure the work climate in EHS. First, based on the Spearman-Brown prediction (or prophecy) formula that relates reliability and length of the test (Brown, 1910; Spearman, 1910), we will estimate the number of items that we can remove in each factor without implying a decrease in the reliability coefficient lower than 0.8, a cut-off point to be considered a good result following the criteria established by George and Mallery (2003). Second, we will develop a Delphi method (Dalkey and Helmer, 1963) to determine, with a sample of at least 20 judges (50% experts in psychometrics and 50% EHS workers), the most redundant items to be removed. In further applications of the resulting short scale, we will check whether, as expected, the reliability coefficient maintains good reliability for coefficients in all the factors. To cover the second limitation (the use of one item as a criterion), we will use another scale as a criterion, specifically the scale proposed by Biggs et al. (2016), which presents partial validity evidence (based on relationships with other variables).

Additionally, we will delve into validity based on relations to other variables that present convergent and discriminant evidence of the resultant scale following the multitrait-multimethod matrix proposed by Campbell and Fiske (1959). We will request completion of three different instruments from ~100 EHS workers: the scale we proposed; another scale with partial validity evidence to measure the work climate in EHS (the one proposed by Biggs et al., 2016); and the Safety Attitudes Questionnaire (Sexton et al., 2016), an instrument that measures safety climate, a construct similar to work climate although not exactly the same (work climate is considered wider). We expect to find high reliability coefficients in the three instruments, a high correlation between the two instruments that measure work climate in EHS (convergent evidence) and a low correlation (at least lower than that obtained in the convergent evidence study) between our instrument and the one that measures a different construct (discriminant evidence).

Finally, we will test its factorial invariance to check whether the use of the scale obtained can be generalized to different hospitals, countries, genders, roles, and professions. First, we will gather a massive number of answers to the scale from participants of different hospitals in one country, from different countries (initially, Spain and Chile), different genders, different roles (coordinator/boss or without being in charge of other people), and different professions (nurses, nursing assistants, doctors, orderlies, and administrative officers). Second, using the software LISREL, we will test the invariance of the structural model and the measurement model across groups (Byrne, 1998).

## Ethics statement

This study was carried out in accordance with the recommendations of the “Declaration on bioethics and human rights, UNESCO, 2005” with written informed consent from all subjects. All subjects gave written informed consent in accordance with the Declaration of Helsinki. The protocol was approved by the “Ethics Committee, Universidad Autónoma de Chile.”

## Author contributions

The initial idea was generated by SC-M and was later supplemented and developed by SS-C, JL-L, and SC-M. JL-L gathered all the information and partially analyzed the data in Studies 1, 2, and 3. FH-T carried out most of the data analyses in Study 3. The manuscript was written by SS-C; JL-L and SC-M made a substantial contribution to the design of the paper, improving both its writing and structure. All gave consent to this final version for publication, and all agreed to be responsible for all aspects of the work, such as the accuracy of the data and the integrity of the research.

### Conflict of interest statement

The authors declare that the research was conducted in the absence of any commercial or financial relationships that could be construed as a potential conflict of interest.
