# Supplementary material for: Development of a Work Climate Scale in Emergency Health Services
Source: Front Psychol. 2018 Jan 22;9:10. doi: 10.3389/fpsyg.2018.00010 (PMC5786539; doi:10.3389/fpsyg.2018.00010)
Supplement: Supplementary file 1 [file Table1.DOCX]

Supplementary Material

Development of a Work Climate Scale in Emergency Health Services

**Susana Sanduvete-Chaves, José A. Lozano-Lozano, Salvador Chacón-Moscoso^*^, Francisco P. Holgado-Tello**

*** Correspondence:** Salvador Chacón-Moscoso: [schacon@us.es](mailto:schacon@us.es)

**Supplementary Table 1**. Items measuring the work climate in emergency health services obtained from in-depth interviews (Lozano-Lozano, Chacón-Moscoso, Sanduvete-Chaves, & Pérez-Gil, J. A., 2013).

| **Family** | **Code** | **Item** |
| --- | --- | --- |
| Experience | Time in the current workplace | 1. We have the necessary experience to do our work well |
| Work conditions | Kind of workday | 2. Our workday is adequate to develop our work |
|  | Adequacy time | 3. We have the necessary time to perform my work well |
|  | Number of users | 4. We have the necessary time to attend to our users |
|  | Comparison with other hospitals | 5. The amount of work I do is similar to what I did in other centers where worked |
| Relations outside the group | Relation with other services | 6. We have good relation with the other services of the center |
|  | Relation with patients | 7. We have a good relationship with our patients |
|  | Relation with relatives | 8. We have a good relationship with the relatives of our patients |
|  | Relation patients and hospital | 9. Our patients have a good relationship with the hospital where we work |
| Infrastructure | Infrastructure | 10. We have the necessary infrastructure to carry out our work |
| Functioning of our service | Training | 11. We receive the necessary training to carry out our work |
|  | Characteristics of our service | 12. The characteristics of our service are appropriate to carry out our work |
|  | Functioning of the service | 13. Our service works correctly |
|  | Productivity/performance | 14. Our work group is known for productivity and high performance |
|  | Protocols for action | 15. Our work is always guided by protocols for action |
| Motivation | Motivation | 16. We feel motivated doing our work |
| Recognition | Group recognition | 17. The merit of our good job is recognized |
|  | Recognition of the profession | 18. Our colleagues value our profession |
|  | Individual recognition | 19. We are appreciated for the work we do |
|  | Recognition of the specialization | 20. Our specialization is recognized by workmates |
| Expectations | Expectations | 21. Our expectations when we entered the working group have been fulfilled |
| Patient adequacy | Kind of patient | 22. The type of patient we serve fits with the specialization of the service |
|  | Patient in emergency | 23. We always attend to patients who come in in a state of emergency |
|  | Knowledge about the patient | 24. We know very well the characteristics that our patients have |
| Coordination/ | Coordination with other services | 25. We coordinate our work with the other hospital services |
| Relations within the group | Communication between members | 26. We have good communication between the members of the work group |
|  | Relations between members | 27. We have good relationship between all the members of the work group |
|  | Comfort between members | 28. I feel comfortable working with the other members of my work group |
|  | Personal relations between members | 29. I have good personal relationships with the other members of the work group |
|  | Work group climate | 30. We work in a good work group climate |
|  | Reasons for entering the group | 31. One of the reasons for entering the group is I already knew some of its members |
|  | Conflict management | 32. We know how to manage the conflict that is generated between us |
|  | Relation with the chief | 33. We have a good relationship with our work group chief |
| Work performance | Individual competencies | 34. I know my professional shortcomings when developing my work |
|  | Functions | 35. We know the functions that each of the members of the work group has |
|  | Type of illness | 36. The type of problems that our patients present fit the specialty of our service |
|  | Group competencies | 37. We know our shortcomings as group in the performance of our work |
|  | Improvement proposals | 38. We are allowed to make proposals to improve our work |
