# Supplementary material for: Development of a Work Climate Scale in Emergency Health Services
Source: Front Psychol. 2018 Jan 22;9:10. doi: 10.3389/fpsyg.2018.00010 (PMC5786539; doi:10.3389/fpsyg.2018.00010)
Supplement: Supplementary file 2 [file DataSheet1.DOCX]

Supplementary Material

Development of a Work Climate Scale in Emergency Health Services

**Susana Sanduvete-Chaves, José A. Lozano-Lozano, Salvador Chacón-Moscoso^*^, Francisco P. Holgado-Tello**

*** Correspondence:** Salvador Chacón-Moscoso: [schacon@us.es](mailto:schacon@us.es)

**Supplementary Data 1**. Items used to measure work climate in emergency health services in Study 1: Identification of the latent conceptual structure of work climate: A mixed-method approach (Spanish version, translated from Perry et al., 2005; the English version can be seen in Table 1).

1. Nos sentimos orgullosos de nuestro trabajo
2. Nuestro grupo de trabajo es conocido por la calidad de su trabajo
3. Tenemos un propósito común
4. Nos esforzamos por entender las necesidades de nuestros usuarios
5. Nos adaptamos fácilmente a nuevas circunstancias
6. Nos esforzamos por lograr resultados exitosos
7. Entendemos la importancia del trabajo de cada miembro del grupo
8. Somos conscientes de las habilidades de cada uno
9. Nos esforzamos por mejorar nuestro desempeño
10. Prestamos atención a cómo de bien trabajamos juntos
11. Se nos reconocen nuestras aportaciones personales
12. Tenemos los recursos necesarios para hacer nuestro trabajo bien
13. Seguimos un plan que guía nuestras actividades
14. Participamos en las decisiones de nuestro grupo de trabajo
15. Nuestro grupo de trabajo es productivo
16. Nuestro trabajo es importante
17. Desarrollamos nuestras habilidades y conocimientos
18. Tenemos claro qué se espera de nuestro trabajo
