# Supplementary material for: Development of a Work Climate Scale in Emergency Health Services
Source: Front Psychol. 2018 Jan 22;9:10. doi: 10.3389/fpsyg.2018.00010 (PMC5786539; doi:10.3389/fpsyg.2018.00010)
Supplement: Supplementary file 3 [file DataSheet2.DOCX]

Supplementary Material

Development of a Work Climate Scale in Emergency Health Services

**Susana Sanduvete-Chaves, José A. Lozano-Lozano, Salvador Chacón-Moscoso^*^, Francisco P. Holgado-Tello**

*** Correspondence:** Salvador Chacón-Moscoso: [schacon@us.es](mailto:schacon@us.es)

**Supplementary Data 2.** Questionnaire to obtain validity evidence based on test content through experts’ judgment in the instrument to delimit the quality of the work climate scale in the emergency health service of a healthcare organization.

Scale to measure the Quality of Work Climate in the Emergency Health Service:

**Study of Validity Evidence of the Resultant Instrument based on Test Content**

**Instructions**. We aim to develop a study to obtain validity evidence based on the test content of this instrument to measure the work climate in emergency health services. As an expert, please evaluate the adequacy of each item with its factor (dimension) from 1 to 5, with 1 being “strongly disagree” and 5 “strongly agree” referring to three different aspects: (a) representativeness (R), referring to the degree to which the item represents its factor; (b) utility (U), referring to the extent to which the item is useful for evaluating the quality of the work climate with respect to its factor; and (c) feasibility (F), referring to whether the data can be gathered. Thank you very much for your collaboration.

| \| Dimension 1. Work satisfaction \| \| --- \| \| 1. We take pride in our work \| \| 2. Our work group is known for quality work \| \| 3. We have a common purpose \| \| 4. We seek to understand the needs of our clients \| \| 5. We readily adapt to new circumstances \| \| 6. We strive to achieve successful outcomes \| \| 7. We have the necessary experience to do our work well \| \| 8. Our workday is adequate to develop our work \| \| 9. We have the necessary time to perform our work well \| \| 10. We have the necessary time to attend to our users \| \| 11. The amount of work I do is similar to the amount I did in other centers where I worked \| \| 12. We have good relations with the other services of the center \| \| 13. We have a good relationship with our patients \| \| 14. We have a good relationship with the relatives of our patients \| \| 15. Our patients have a good relationship with the hospital where we work \| \| Dimension 2. Productivity/achievement of aims \| \| 16. We understand the relevance of the job of each member in our group \| \| 17. We understand each other’s capabilities \| \| 18. We strive to improve our performance \| \| 19. We pay attention to how well we are working together \| \| 20. We have the necessary infrastructure to carry out our work \| \| 21. We receive the necessary training to carry out our work \| \| 22. The characteristics of our service are appropriate to carry out our work \| \| 23. Our service works correctly \| \| 24. Our work group is known for productivity and high performance \| \| 25. Our work is always guided by protocols for action \| \| 26. We feel motivated doing our work \| \| 27. The merit of our good job is recognized \| \| 28. Our colleagues value our profession \| \| 29. We are appreciated for the work we do \| \| 30. Our specialization is recognized by workmates \| \| 31. Our expectations when we entered the working group have been fulfilled \| \| 32. The type of patient we serve fits with the specialization of the service \| \| 33. We always attend to patients who come into in a state of emergency \| \| 34. We know very well the characteristics that our patients have \| \| 35. We coordinate our work with the other hospital services \| \| Dimension 3. Interpersonal relationships \| \| 36. We are recognized for our individual contributions \| \| 37. We have the resources we need to do our jobs well \| \| 38. We have a plan that guides our activities \| \| 39. We participate in the decisions of our work group \| \| 40. Our work group is productive \| \| 41. We have good communication between the members of the work group \| \| 42. We have good relationships between all the members of the work group \| \| 43. I feel comfortable working with the other components of my work group \| \| 44. I have good personal relationships with the other members of the work group \| \| 45. We work in a good work group climate \| \| 46. One of the reasons for entering the work group is that I already knew some of its members \| \| 47. We know how to manage the conflict that is generated between us \| \| 48. We have a good relationship with our work group chief \| \| Dimension 4. Performance at work \| \| 49. Our work is important \| \| 50. We develop our skills and knowledge \| \| 51. We are clear about what is expected in our work \| \| 52. I know my professional shortcomings when developing my work \| \| 53. We know the functions that each of the members of the work group has \| \| 54. The type of problems that our patients present fit the specialty of our service \| \| 55. We know our shortcomings as group in the performance of our work \| \| 56. We are allowed to make proposals to improve our work \| |
| --- | --- | --- | --- | --- | --- | --- | --- | --- | --- | --- | --- | --- | --- | --- | --- | --- | --- | --- | --- | --- | --- | --- | --- | --- | --- | --- | --- | --- | --- | --- | --- | --- | --- | --- | --- | --- | --- | --- | --- | --- | --- | --- | --- | --- | --- | --- | --- | --- | --- | --- | --- | --- | --- | --- | --- | --- | --- | --- | --- | --- |

Please add any comments you consider important to improve this work climate scale in emergency health services:
