# Supplementary material for: Development of a Work Climate Scale in Emergency Health Services
Source: Front Psychol. 2018 Jan 22;9:10. doi: 10.3389/fpsyg.2018.00010 (PMC5786539; doi:10.3389/fpsyg.2018.00010)
Supplement: Supplementary file 4 [file DataSheet3.DOCX]

Supplementary Material

Development of a Work Climate Scale in Emergency Health Services

**Susana Sanduvete-Chaves, José A. Lozano-Lozano, Salvador Chacón-Moscoso^*^, Francisco P. Holgado-Tello**

*** Correspondence:** Salvador Chacón-Moscoso: [schacon@us.es](mailto:schacon@us.es)

**Supplementary Data 3.** Questionnaire to obtain validity evidence based on test content through experts’ judgment in the instrument to delimit the quality of the work climate scale in the emergency health service of a healthcare organization (Spanish version).

Escala para medir la Calidad del Clima Laboral en el Servicio de Urgencias:

**Estudio de Evidencias de Validez en base al Contenido del Instrumento**

**Instrucciones**. Nuestro objetivo es llevar a cabo un estudio para obtener evidencias de validez en base al contenido de este instrumento para medir el clima laboral en los servicios de urgencias. Como experto, por favor evalúe la adecuación de cada ítem a su dimensión de 1 a 5, siendo 1 totalmente en desacuerdo y 5 totalmente de acuerdo, en relación a tres aspectos: (a) representatividad, refiriéndose al grado en que el ítem representa su dimensión; (b) utilidad, refiriéndose al grado en que el ítem es útil para evaluar la calidad del clima laboral con respecto a su dimensión; y c) viabilidad, haciendo referencia a si las circunstancias hacen posible la existencia y recogida de la información que se pretende obtener con el ítem. Muchas gracias por su colaboración.

| Dimensión 1. Satisfacción con el trabajo |
| --- |
| 1. Nos sentimos orgullosos de nuestro trabajo |
| 2. Nuestro grupo de trabajo es conocido por la calidad de su trabajo |
| 3. Tenemos un propósito común |
| 4. Nos esforzamos por entender las necesidades de nuestros usuarios |
| 5. Nos adaptamos fácilmente a nuevas circunstancias |
| 6. Nos esforzamos por lograr resultados exitosos |
| 7. Tenemos la experiencia necesaria para realizar bien nuestro trabajo |
| 8. Nuestra jornada laboral es adecuada para desarrollar nuestro trabajo |
| 9. Dispongo del tiempo necesario para realizar bien mi trabajo |
| 10. Disponemos del tiempo necesario para atender a nuestros usuarios |
| 11. La cantidad de trabajo que realizo es similar a la que tuve en otros centros donde he trabajado |
| 12. Tenemos buena relación con los demás servicios del centro |
| 13. Tenemos buena relación con nuestros pacientes |
| 14. Tenemos buena relación con los familiares de nuestros pacientes |
| 15. Nuestros pacientes tienen buena relación con el hospital donde trabajamos |
| Dimensión 2. Productividad/ Logro de objetivos |
| 16. Entendemos la importancia del trabajo de cada miembro del grupo |
| 17. Somos conscientes de las habilidades de cada uno |
| 18. Nos esforzamos por mejorar nuestro desempeño |
| 19. Prestamos atención a cómo de bien trabajamos juntos |
| 20. Contamos con los recursos y la infraestructura necesaria para realizar nuestro trabajo |
| 21. Recibimos la formación necesaria para realizar nuestro trabajo |
| 22. Las características de nuestro servicio son las apropiadas para desempeñar nuestro trabajo |
| 23. Nuestro servicio funciona correctamente |
| 24. Se conoce a nuestro grupo de trabajo por nuestra productividad y alto rendimiento |
| 25. Nuestro trabajo siempre es guiado por unos protocolos de actuación |
| 26. Nos sentimos motivados realizando nuestro trabajo |
| 27. Se nos reconoce lo bien que realizamos nuestro trabajo |
| 28. Nuestros compañeros valoran nuestra profesión |
| 29. Se nos valora el trabajo que realizamos |
| 30. Nuestra especialización es reconocida por los compañeros |
| 31. Nuestras expectativas cuando entramos al grupo de trabajo se han cumplido |
| 32. El tipo de paciente al que atendemos se ajusta a la especialización del servicio |
| 33. Siempre atendemos pacientes que vienen en estado de emergencia |
| 34. Conocemos muy bien las características que tienen nuestros pacientes |
| 35. Coordinamos nuestro trabajo con los demás servicios del hospital |
| Dimensión 3. Relaciones interpersonales |
| 36. Se nos reconocen nuestras aportaciones personales |
| 37. Tenemos los recursos necesarios para hacer nuestro trabajo bien |
| 38. Seguimos un plan que guía nuestras actividades |
| 39. Participamos en las decisiones de nuestro grupo de trabajo |
| 40. Nuestro grupo de trabajo es productivo |
| 41. Tenemos buena comunicación entre los miembros del grupo de trabajo |
| 42. Tenemos buena relación entre todos los miembros del grupo de trabajo |
| 43. Me siento a gusto trabajando con los demás componentes de mi grupo de trabajo |
| 44. Mantengo buenas relaciones personales con los demás miembros de trabajo |
| 45. Trabajamos en un buen clima de grupo de trabajo |
| 46. Uno de los motivos de entrar al grupo de trabajo es que ya conocía a alguno de sus miembros |
| 47. Sabemos gestionar el conflicto que se genera entre nosotros |
| 48. Tenemos buena relación con nuestro director de grupo de trabajo |
| Dimensión 4. Rendimiento en el trabajo |
| 49. Nuestro trabajo es importante |
| 50. Desarrollamos nuestras habilidades y conocimientos |
| 51. Tenemos claro qué se espera de nuestro trabajo |
| 52. Conozco las carencias profesionales que tengo al desarrollar mi trabajo |
| 53. Conocemos las funciones que tienen cada uno de los miembros del grupo de trabajo |
| 54. El tipo de problemática que presentan nuestros pacientes se ajusta a la especialidad de nuestro servicio |
| 55. Conocemos nuestras carencias como grupo a la hora de desempeñar nuestro trabajo |
| 56. Se nos permite hacer propuestas para mejorar nuestro trabajo |

Por favor, añada a continuación cualquier comentario que considere de interés para mejorar esta escala de clima laboral en el servicio de urgencias:
