# Supplementary material for: Development of a Work Climate Scale in Emergency Health Services
Source: Front Psychol. 2018 Jan 22;9:10. doi: 10.3389/fpsyg.2018.00010 (PMC5786539; doi:10.3389/fpsyg.2018.00010)
Supplement: Supplementary file 5 [file DataSheet4.DOCX]

Supplementary Material

Development of a Work Climate Scale in Emergency Health Services

**Susana Sanduvete-Chaves, José A. Lozano-Lozano, Salvador Chacón-Moscoso^*^, Francisco P. Holgado-Tello**

*** Correspondence:** Salvador Chacón-Moscoso: [schacon@us.es](mailto:schacon@us.es)

**Supplementary Data 4.** Work Climate Scale in Emergency Health Services: final printable version ready to be used (authors give their permission for its use if this work is correctly referenced) and instructions to obtain participants’ scores.

**Work Climate Scale in Emergency Health Services**

Instructions for participants: this questionnaire aims to collect relevant information about the work climate in the emergency health services of this hospital. The information requested will be anonymous and used exclusively for the task we are carrying out on work climate quality, so its content is private. To guarantee the confidentiality of your answers, this information will never be identified with the person who gave the answers. Please read the questions carefully and respond honestly **from 1 (strongly disagree) to 5 (strongly agree)**. Once the information is collected, you will have access to the data if you wish. We thank you in advance for your time and collaboration in this task.

| **Factor 1. Work satisfaction** | **Score** |
| --- | --- |
| 1. We take pride in our work | 1 2 3 4 5 |
| 2. We seek to understand the needs of our clients | 1 2 3 4 5 |
| 3. We readily adapt to new circumstances | 1 2 3 4 5 |
| 4. We strive to achieve successful outcomes | 1 2 3 4 5 |
| 5. We have the necessary experience to do our work well | 1 2 3 4 5 |
| 6. Our workday is adequate to develop our work | 1 2 3 4 5 |
| 7. We have good relations with the other services of the center | 1 2 3 4 5 |
| 8. We understand the relevance of the job of each member in our group | 1 2 3 4 5 |
| 9. Our work is important | 1 2 3 4 5 |
| 10. We develop our skills and knowledge | 1 2 3 4 5 |
| **Factor 2. Productivity/achievement of aims** | **Score** |
| 11. Our work group is known for quality work | 1 2 3 4 5 |
| 12. We have a common purpose | 1 2 3 4 5 |
| 13. We have the necessary infrastructure to carry out our work | 1 2 3 4 5 |
| 14. We receive the necessary training to carry out our work | 1 2 3 4 5 |
| 15. The characteristics of our service are appropriate to carry out our work | 1 2 3 4 5 |
| 16. Our service works correctly | 1 2 3 4 5 |
| 17. Our work group is known for productivity and high performance | 1 2 3 4 5 |
| 18. We feel motivated doing our work | 1 2 3 4 5 |
| 19. The merit of our good job is recognized | 1 2 3 4 5 |
| 20. Our colleagues value our profession | 1 2 3 4 5 |
| 21. We are appreciated for the work we do | 1 2 3 4 5 |
| 22. Our specialization is recognized by workmates | 1 2 3 4 5 |
| 23. Our expectations when we entered the working group have been fulfilled | 1 2 3 4 5 |
| 24. The type of patient we serve fits with the specialization of the service | 1 2 3 4 5 |
| 25. We know very well the characteristics that our patients have | 1 2 3 4 5 |
| 26. We coordinate our work with the other hospital services | 1 2 3 4 5 |
| 27. We are recognized for our individual contributions | 1 2 3 4 5 |
| 28. We have a plan that guides our activities | 1 2 3 4 5 |
| 29. We participate in the decisions of our work group | 1 2 3 4 5 |
| 30. We are clear about what is expected in our work | 1 2 3 4 5 |
| **Factor 3. Interpersonal relationships** | **Score** |
| 31. We have good communication between the members of the work group | 1 2 3 4 5 |
| 32. We have good relationships between all the members of the work group | 1 2 3 4 5 |
| 33. I feel comfortable working with the other components of my work group | 1 2 3 4 5 |
| 34. I have good personal relationships with the other members of the work group | 1 2 3 4 5 |
| 35. We work in a good work group climate | 1 2 3 4 5 |
| 36. We understand each other’s capabilities | 1 2 3 4 5 |
| **Factor 4. Performance at work** | **Score** |
| 37. I know my professional shortcomings when developing my work | 1 2 3 4 5 |
| 38. We know the functions that each of the members of the work group has | 1 2 3 4 5 |
| 39. The type of problems that our patients present fit the specialty of our service | 1 2 3 4 5 |
| 40. We know our shortcomings as group in the performance of our work | 1 2 3 4 5 |

Instructions for evaluators: a total score can be obtained by adding the scores given in each item (with 40 being the lowest possible score and 200 the highest possible). In the same way, a score for each factor can be obtained by adding their corresponding items.
