# Supplementary material for: Development of a Work Climate Scale in Emergency Health Services
Source: Front Psychol. 2018 Jan 22;9:10. doi: 10.3389/fpsyg.2018.00010 (PMC5786539; doi:10.3389/fpsyg.2018.00010)
Supplement: Supplementary file 6 [file DataSheet5.DOCX]

Supplementary Material

Development of a Work Climate Scale in Emergency Health Services

**Susana Sanduvete-Chaves, José A. Lozano-Lozano, Salvador Chacón-Moscoso^*^, Francisco P. Holgado-Tello**

*** Correspondence:** Salvador Chacón-Moscoso: [schacon@us.es](mailto:schacon@us.es)

**Supplementary Data 5.** Work Climate Scale in Emergency Health Services: final printable version ready to be used (authors give their permission for its use if this work is correctly referenced) and instructions to obtain participants’ scores (Spanish version).

**Escala de Clima Laboral para Servicios de Emergencia**

Instrucciones para los participantes: con este cuestionario se pretende recoger información relevante acerca del clima laboral en los servicios de Urgencia, Unidad de Cuidados Intensivos y Dispositivos de Cuidados Críticos y Urgencias de este hospital. La información que se le pide es de uso exclusivo y anónimo para la tarea que estamos llevando a cabo sobre la calidad del clima laboral, por lo que su contenido es totalmente privado. Con el fin de garantizar la confidencialidad de sus respuestas, en ningún momento posterior se identificará esta información con la persona que la emite. Le rogamos que lea con detenimiento las preguntas y las responda sinceramente según su opinión, **valorando de 1, totalmente en desacuerdo a 5, totalmente de acuerdo**. Una vez recogida la información, tendrá acceso a los datos obtenidos si así lo desea. Le agradecemos de antemano su tiempo y colaboración en esta tarea.

| **Factor 1. Satisfacción con el trabajo** | **Puntuación** |
| --- | --- |
| 1. Nos sentimos orgullosos de nuestro trabajo | 1 2 3 4 5 |
| 2. Nos esforzamos por entender las necesidades de nuestros usuarios | 1 2 3 4 5 |
| 3. Nos adaptamos fácilmente a nuevas circunstancias | 1 2 3 4 5 |
| 4. Nos esforzamos por lograr resultados exitosos | 1 2 3 4 5 |
| 5. Tenemos la experiencia necesaria para realizar bien nuestro trabajo | 1 2 3 4 5 |
| 6. Nuestra jornada laboral es adecuada para desarrollar nuestro trabajo | 1 2 3 4 5 |
| 7. Tenemos buena relación con los demás servicios del centro | 1 2 3 4 5 |
| 8. Entendemos la importancia del trabajo de cada miembro del grupo | 1 2 3 4 5 |
| 9. Nuestro trabajo es importante | 1 2 3 4 5 |
| 10. Desarrollamos nuestras habilidades y conocimientos | 1 2 3 4 5 |
| **Factor 2. Productividad/ Logro de objetivos** | **Puntuación** |
| 11. Nuestro grupo de trabajo es conocido por la calidad de su trabajo | 1 2 3 4 5 |
| 12. Tenemos un propósito común | 1 2 3 4 5 |
| 13. Contamos con los recursos y la infraestructura necesaria para realizar nuestro trabajo | 1 2 3 4 5 |
| 14. Recibimos la formación necesaria para realizar nuestro trabajo | 1 2 3 4 5 |
| 15. Las características de nuestro servicio son las apropiadas para desempeñar nuestro trabajo | 1 2 3 4 5 |
| 16. Nuestro servicio funciona correctamente | 1 2 3 4 5 |
| 17. Se conoce a nuestro grupo de trabajo por nuestra productividad y alto rendimiento | 1 2 3 4 5 |
| 18. Nos sentimos motivados realizando nuestro trabajo | 1 2 3 4 5 |
| 19. Se nos reconoce lo bien que realizamos nuestro trabajo | 1 2 3 4 5 |
| 20. Nuestros compañeros valoran nuestra profesión | 1 2 3 4 5 |
| 21. Se nos valora el trabajo que realizamos | 1 2 3 4 5 |
| 22. Nuestra especialización es reconocida por los compañeros | 1 2 3 4 5 |
| 23. Nuestras expectativas cuando entramos al grupo de trabajo se han cumplido | 1 2 3 4 5 |
| 24. El tipo de paciente al que atendemos se ajusta a la especialización del servicio | 1 2 3 4 5 |
| 25. Conocemos muy bien las características que tienen nuestros pacientes | 1 2 3 4 5 |
| 26. Coordinamos nuestro trabajo con los demás servicios del hospital | 1 2 3 4 5 |
| 27. Se nos reconocen nuestras aportaciones personales | 1 2 3 4 5 |
| 28. Seguimos un plan que guía nuestras actividades | 1 2 3 4 5 |
| 29. Participamos en las decisiones de nuestro grupo de trabajo | 1 2 3 4 5 |
| 30. Tenemos claro qué se espera de nuestro trabajo | 1 2 3 4 5 |
| **Factor 3. Relaciones interpersonales** | **Puntuación** |
| 31. Tenemos buena comunicación entre los miembros del grupo de trabajo | 1 2 3 4 5 |
| 32. Tenemos buena relación entre todos los miembros del grupo de trabajo | 1 2 3 4 5 |
| 33. Me siento a gusto trabajando con los demás componentes de mi grupo de trabajo | 1 2 3 4 5 |
| 34. Mantengo buenas relaciones personales con los demás miembros de trabajo | 1 2 3 4 5 |
| 35. Trabajamos en un buen clima de grupo de trabajo | 1 2 3 4 5 |
| 36. Somos conscientes de las habilidades de cada uno | 1 2 3 4 5 |
| **Factor 4. Rendimiento en el trabajo** | **Puntuación** |
| 37. Conozco las carencias profesionales que tengo al desarrollar mi trabajo | 1 2 3 4 5 |
| 38. Conocemos las funciones que tienen cada uno de los miembros del grupo de trabajo | 1 2 3 4 5 |
| 39. El tipo de problemática que presentan nuestros pacientes se ajusta a la especialidad de nuestro servicio | 1 2 3 4 5 |
| 40. Conocemos nuestras carencias como grupo a la hora de desempeñar nuestro trabajo | 1 2 3 4 5 |

Instrucciones para los evaluadores: se puede obtener una puntuación global sumando las puntuaciones dadas a cada ítem (siendo 40 la puntuación más baja posible y 200 la más alta). De igual manera, se puede obtener una puntuación para cada factor sumando sus ítems correspondientes.
